# Coherent Polaritons in WSe_2_‑Monolayer-Sandwiched Au-Nanodisk-on-Mirror Structures

**DOI:** 10.1021/acsnano.5c06093

**Published:** 2025-07-04

**Authors:** He Huang, Bo Tian, Yang Chen, Xinyue Xia, Ximin Cui, Lei Shao, Huanjun Chen, Jianfang Wang

**Affiliations:** † Department of Physics, 26451 The Chinese University of Hong Kong, Shatin, Hong Kong SAR 999077, China; ‡ State Key Laboratory of Optoelectronic Materials and Technologies, Guangdong Provincial Key Laboratory of Display Materials and Technologies, School of Electronics and Information Technology, 26469 Sun Yat-sen University, Guangzhou, Guangdong 510275, China; § State Key Laboratory of Radio Frequency Heterogeneous Integration, College of Electronics and Information Engineering, 47890 Shenzhen University, Shenzhen, Guangdong 518060, China

**Keywords:** plasmon resonance, particle-on-mirror structure, transition metal dichalcogenide, exciton, strong coupling, valley polarization, valley coherence

## Abstract

Owing to their direct bandgap and spin-valley locking in the visible to near-infrared region, transition metal dichalcogenide monolayers have emerged as promising candidates for next-generation atomically thin optoelectronics. The locking of the electron spin to different valleys offers a valley degree of freedom for information encoding. Nevertheless, the ability to address valley coherence is an important precondition for achieving the quantum manipulation of the valley index. Current access and control of coherent valley information typically relies on the use of high-quality distributed Bragg reflectors and extreme conditions involving an operating temperature of ∼4 K and an applied magnetic field up to 9 T, which is far from the demands of quantum optics and practical optoelectronic applications. In this work, we demonstrate valley coherence under ambient conditions within a sub-145 nm wide area through strong plasmon–exciton coupling featured by distinct Rabi splitting in the photoluminescence spectra. These observations provide insights into strong plasmon–exciton coupling and practical valleytronics.

## Graphical abstract

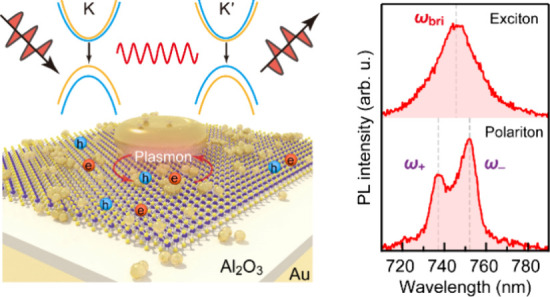

The ever-increasing demand for computing power drives the continuous miniaturization and densification of computational elements, pushing the traditional silicon-based electronics to their operation limit. Apart from their charges, electrons demonstrate supplementary degrees of freedom such as spin and valley, which can be leveraged for efficient information encoding, storage, and processing. Over the past several decades, significant progress has been made in manipulating electron spin for semiconductor spintronic devices.^1,2^ However, experimental advancements toward the control of the valley degree of freedom have been constrained until the emergence of two-dimensional (2D) materials with honeycomb structures.^3,4^ These materials, such as graphene sheets with broken inversion symmetry and transition metal dichalcogenide (TMDC) monolayers, possess two degenerate and inequivalent valleys (K and K′) at the corners of the first Brillouin zone,^5^ which endows valley-pseudospins to electrons. To make use of the valley degree of freedom, an essential step is to identify a process in which valley charge carriers respond differently to external stimuli, thereby producing a valley polarization. Schemes based on magnetic, electric, and mechanical means have been proposed to realize the manipulation of the valley index in semimetals.^6,7^ In contrast to semimetals, TMDC monolayers have direct bandgaps in the visible to near-infrared region and inherently lack inversion symmetry.^8^ Their charge carriers in the K and K′ valleys are subject to opposite Berry curvatures and orbital magnetic moments, which give rise to a valley contrasting optical selection rule for interband transitions.^9^ This effect enables the valley-dependent interplay of electrons with different circularly polarized light, providing a robust optical approach for information storage and readout. This intrinsic ability to generate circularly polarized light based on valley-selective excitation is a fundamental advantage. It bridges the photon circular polarization, the electron valley (momentum) and the electron spin, offering a possibility to design electronic devices that can manipulate the spin and momentum of electrons by optical excitation.^10,11^ Compared with conventional emitters such as molecular aggregates and quantum dots, atomically thin 2D TMDCs can be transferred or directly grown to conformally integrate with other optical elements for the development of advanced optoelectronic devices. Their excitonic states can also be engineered through twisted stacking. Strategically designed stacking configurations in these materials allow their excitonic states to be engineered, which leads to new properties and functionalities.^12^ Furthermore, TMDC monolayers possess significant potential in qubit hosting and manipulation,^13^ attracting extensive research in the fields of nanophotonics, valleytronics, and quantum information science.

The ability to address valley coherence is a crucial precondition for achieving valley information processing.^14^ However, valley coherence degenerates rapidly on the order of a few picoseconds in TMDC monolayers.^15^ The access and control of valley coherence typically rely on the use of high-quality distributed-Bragg-reflector (DBR) microcavities to form long-lifetime valley-information-carrying exciton polaritons^16–18^ and extreme conditions that involve an operating temperature at ∼4 K and an applied magnetic field up to 9 T.^16,19^ The coherent manipulation of valley pseudospins at room temperature faces significant hurdles, including phonon-mediated intervalley scattering processes, electron–hole interactions, and the Maialle–Silva–Sham mechanism.^17,20,21^ A prospective strategy for addressing this challenge is to create half-light, half-matter exciton polaritons in TMDC monolayers. Such exciton polaritons inherit valley information but are immune to decoherence processes in TMDC monolayers because of extra decay paths introduced by exciton–cavity coupling, which is critical for retaining valley coherence at elevated temperatures. On the other hand, the use of DBRs is difficult to scale down the mode volume and reduce the number of excitons contributing to the coupling down to one, as is demanded for quantum optics applications.^22,23^

An alternative approach is to use single plasmonic nanoresonators to form strong plasmon–exciton coupling. Localized surface plasmon resonance possesses the superior ability to concentrate electromagnetic field onto the subwavelength scale, which can effectively reduce the mode volume and effective exciton number. A recent work on the strong coupling between a WS_2_ monolayer and a nanoprism–film gap resonator demonstrated a successful decrease in the effective exciton number down to ∼2, which represents an essential step forward toward the achievement of quantum manipulation of TMDC excitons.^24^ However, despite the intense investigation on the strong coupling in TMDC layers with single plasmonic nanoresonators, most published works have failed to observe the characteristic signature of exciton polaritons, the splitting of the photoluminescence (PL) peak into two distinct maxima corresponding to the upper and lower polaritonic energies.^25–27^ Only split-peak spectra in dark-field scattering measurements have been acquired and interpreted. Unlike PL, scattering spectra can show two maxima in the intermediate regime because of Fano-like interferences between plasmons and emitter dipoles,^28^ making the regime difficult to distinguish if only scattering is considered. On the other hand, PL as an incoherent process undergoes peak splitting only in the strong coupling regime.^28,29^ Splitting in the PL spectrum has therefore been recognized as the definitive signature of Rabi splitting. To access coherent valley emissions, it is important to acquire PL emissions from exciton polaritons, with minimal interference from degenerated emissions, which however typically dominate under ambient conditions. Valley coherence within a subwavelength lateral dimension has so far not been achieved in TMDC monolayers.

In this work, we demonstrate valley coherence at room temperature within a sub-145 nm wide area through strong plasmon–exciton coupling featured by distinct Rabi splitting in the PL spectra. The resonance wavelength of our WSe_2_-monolayer-sandwiched Au-nanodisk-on-mirror (NDoM) structures is modulated by adjusting the ND diameter from 100 to 145 nm. The peak intensity ratio of the higher-to-lower polaritonic states in the PL spectra is tuned from 0.68 to 1.22 as the plasmon resonance energy is red-shifted from above to below the exciton emission energy. A linear polarization degree of 11.6 ± 2.4% is observed in the PL emissions at room temperature. Co-linearly polarized PL emissions with the excitation light at different incident polarization angles are observed, suggesting intervalley phase coherence. The cavity plasmon mode of our structures has the topological stability of a skyrmion, allowing spatial-perturbation-insensitive efficient coupling between the plasmon resonance and TMDC excitons with varying emission dipole orientations. It is noteworthy that the observation of distinct PL peak splitting benefits from our post structural treatment and polarization-resolved PL measurements. These observations provide insights into the generation of exciton polaritons at the single-nanoparticle level and the manipulation of the valley degree of freedom.

## Results and Discussion

### WSe_2_-Monolayer-Sandwiched Au NDoM Cavities

The plasmonic NDs used in this study were synthesized by seed-mediated growth.^30,31^ Their pseudo-2D geometry, high crystallinity, and size uniformity (Figures 1a and S1) benefit the generation of toroidal plasmon modes for the following spectroscopic studies. Au NDs with an average thickness of 53 ± 1 nm and diameters of 100–145 nm were synthesized. To prevent PL quenching caused by direct contact between the Au film and WSe_2_ monolayer, we prepared a 2.4 nm-thick Al_2_O_3_ spacer layer on the Au film through atomic layer deposition (ALD). WSe_2_-monolayer-sandwiched NDoM cavities were fabricated by first transferring a piece of mechanically exfoliated WSe_2_ monolayer (Figure S2) onto the Au/Al_2_O_3_ substrate and then transferring the as-prepared Au NDs onto the top of the WSe_2_ monolayer (Figure 1b–d). The obtained sample was immersed in ethanol to remove the residual surfactant molecules on the ND surface. The far-field scattering pattern of an individual NDoM cavity displays a discernible red halo surrounding the central green spot (Figure 1c). Scanning electron microscopy (SEM) imaging of an as-prepared device revealed that the transferred WSe_2_ monolayer is sandwiched between the well separated circular NDs and the Au film (Figure 1d). In order to obtain PL signals originating from the nanocavities, we introduced a layer of ultrasmall Au nanoparticles with an average diameter of ∼3 nm on top of the device to suppress the radiative decay of excitons outside the cavity (Figures 1e and S3, S4). When excited with an overbandgap and linearly polarized laser (1.96 eV), excitons in the K and K′ valleys get stimulated. Strong plasmon–exciton coupling results in the generation of exciton polaritons that carry valley information. We refer to ‘valleys’ to describe the K and K′ excitonic components of the polaritons. The plasmonic nanocavity enables a rapid population relaxation of the quasiparticles (excitons or polaritons) in the two valleys. Some of the quasiparticles decay to superimposed right-handed circularly polarized (RCP) (σ+) and left-handed circularly polarized (LCP) (σ−) photons before their phase relation decoheres, giving rise to PL emissions that are colinearly polarized with the excitation light (Figure 1f).

**Figure 1:**
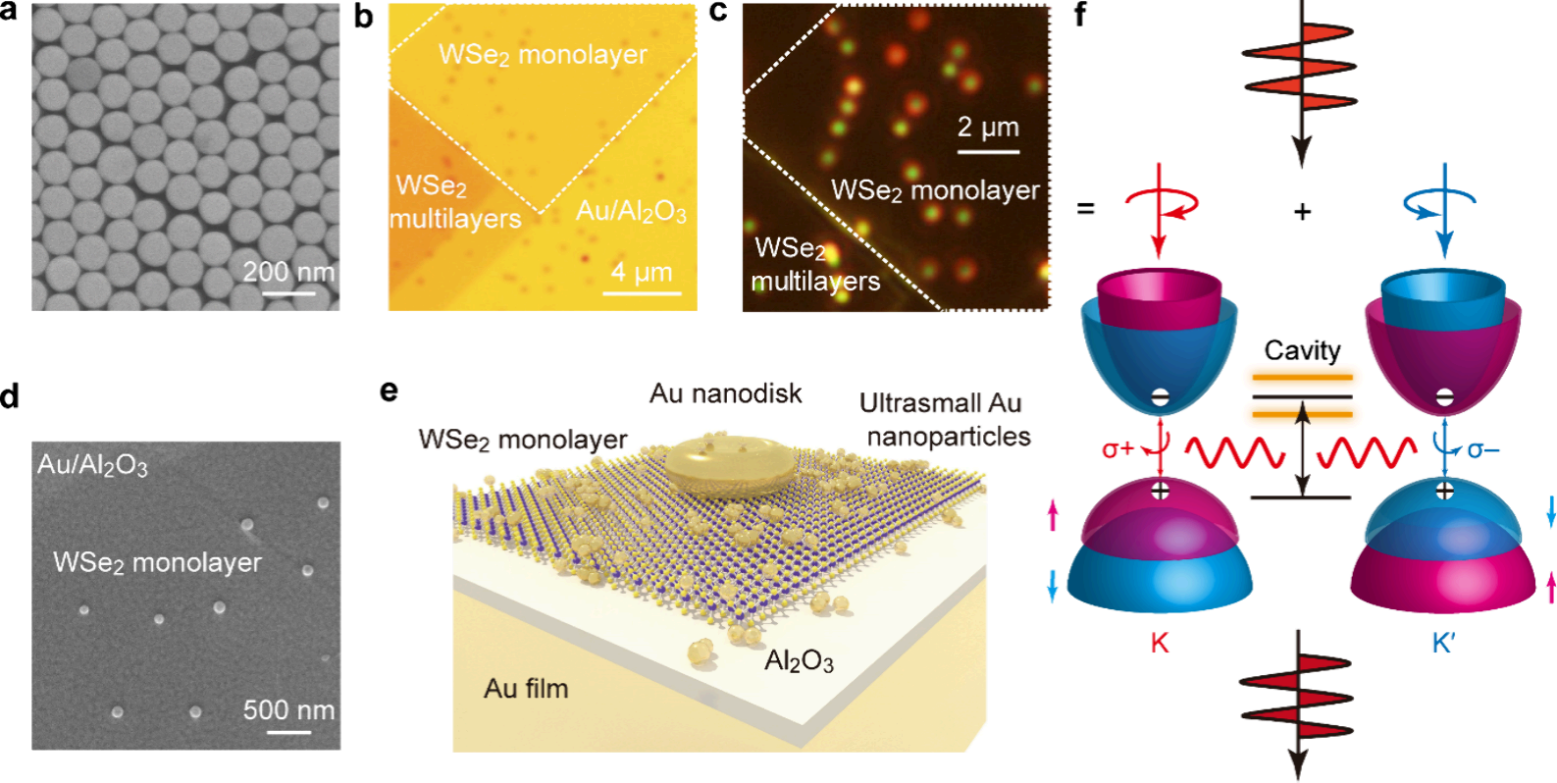
Device architecture and working principle of the cavity-enhanced valley coherence. (a) SEM image of a representative Au ND sample used for the construction of the NDoM cavities. The average diameter and thickness of the displaced Au NDs are 130 ± 8 and 53 ± 1 nm, respectively. (b–d) Optical bright-field (b) and dark-field (c) microscopy images and SEM image (d) of WSe_2_-monolayer-sandwiched NDoM cavities, respectively. The white dashed line indicates the edge of the WSe_2_ monolayer. (e) Schematic showing the architecture of an as-prepared NDoM cavity and deposited ultrasmall Au nanoparticles. (f) Schematic illustrating the formation of coherent exciton polariton states. The excitation light is linearly polarized, which can be decomposed into coherently superimposed RCP light (σ+) and LCP light (σ−) to excite the K and K′ valley excitons of a WSe_2_ monolayer, respectively.

The scattering spectra of the individual nanocavities exhibit an asymmetric scattering peak accompanied by a sudden turning at the short wavelength side of the peak (Figure 2a,b). Based on our previous study,^32^ when the NDoM cavity is excited by out-of-plane polarized light, the Au ND strongly interacts with the substrate and produces out-of-plane electric dipole and toroidal resonances in the gap between the Au ND and the Au film. The scattering peaks can be attributed to toroidal plasmon resonances, where two electric current loops with opposite directions appear near the disk edge at each cross-section plane perpendicular to the substrate. The inflection point is a consequence of the destructive interference between the toroidal plasmon resonance and an out-of-plane dipole plasmon mode, which produces a nonradiating anapole state.^33,34^ More details can be found in our previous work for this type of plasmonic nanoparticle-on-mirror cavity.^32^ We tuned the energy of the cavity mode by varying the ND diameter. When the disk diameter exceeds 135 nm, the first-order toroidal mode redshifts beyond 900 nm while the second-order toroidal mode dominates in the visible region.^32^ By aligning the toroidal mode with the bright A exciton transition energy of WSe_2_ monolayer at ∼751 nm, we resonantly enhanced the radiative decays of K and K′ valley excitons excited by a linearly polarized laser beam, which increased the PL intensity by 2 orders of magnitude (Figure S5a,b). A reduction in the full width at half-maximum of the bright A exciton PL signals was observed when the toroidal resonance was aligned with the bright A exciton transition energy (Figure S5c). As we continued to redshift the toroidal mode beyond the bright A exciton emission wavelength, an additional PL peak emerged at approximately 777 nm (Figure S5a), about 57.0 meV below the bright A exciton photon energy (Figure S5d), which falls within the energy range of dark A excitons in the WSe_2_ monolayer.^35–39^

**Figure 2:**
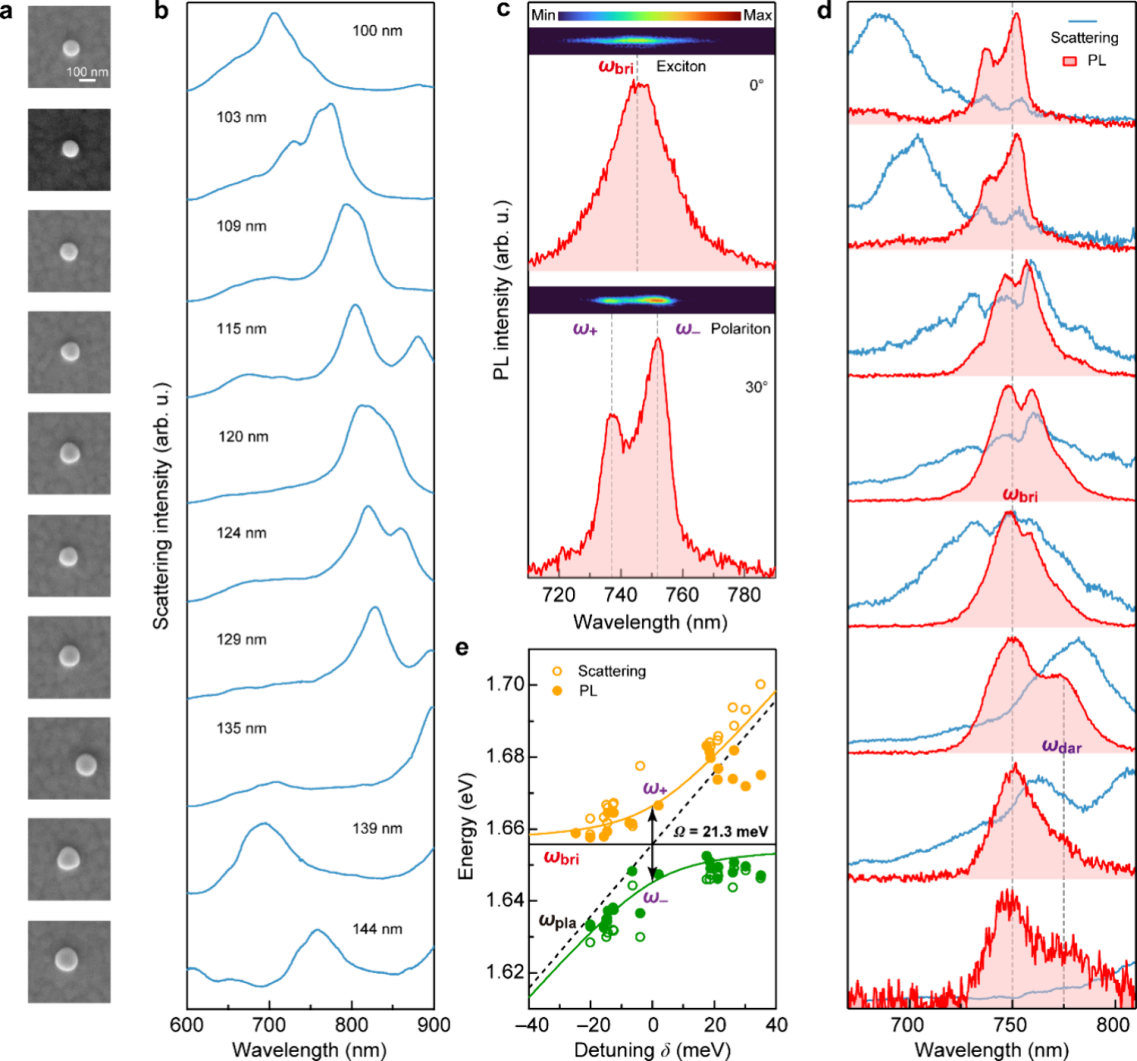
Strong plasmon–exciton coupling in the WSe_2_-monolayer-sandwiched NDoM cavities. (a) SEM images of NDoM cavities with ND diameters ranging from 100 to 144 nm. (b) Measured scattering spectra of the NDoM cavities in (a). (c) PL spectra acquired at detection polarization angles of 0° (upper) and 30° (lower) with an excitation polarization angle of 150°. The insets show the intensity profiles of the PL outputs observed on the charge-coupled camera device as a function of wavelength. They reveal one (upper) and two (lower) maxima at different detection polarization angles. (d) Normalized scattering and PL spectra of the individual NDoM cavities. The PL spectra were measured at detection polarization angles that produced the most pronounced double-peaked spectral features or dark A exciton emissions. The dark A exciton (ω_dar_) and bright A exciton (ω_bri_) energies are indicated in the plot. (e) Extracted anticrossing curve of polaritons. The solid and hollow circles are the polariton energies retrieved from measured PL and scattering spectra through Gaussian fitting, respectively. The two solid lines in orange and green were obtained from fitting according to the coupled-oscillator model. The solid and dashed lines in black represent the uncoupled bright A exciton and plasmon energies, respectively.

### Polarization-Dependent Rabi Splitting of the Photoluminescence

The valley coherence of bright A excitons was investigated by performing emission-polarization-resolved PL measurements, which revealed PL splitting in our (WSe_2_ monolayer)–(NDoM cavity) coupled system. Specifically, in the polarization-resolved PL spectra, we identified in many samples two distinct maxima at certain detection polarization angles (Figure 2c lower panel and Figure S6a), both of which do not coincide with the bright A exciton energy. The PL peak splitting became less pronounced or even completely disappeared as the examined emission polarization varied (Figure 2c upper panel and Figure S6a). These results suggest varying coupling strengths between the plasmon resonance and TMDC A excitons with different emission dipole orientations in the nanocavity. We also observed in the polarization-resolved scattering spectra of the same nanocavities the presence of two small peaks that correspond to the locations of the two maxima in the PL spectra with slightly larger energy splitting (Figure S6b). The peak splitting in the polarization-resolved scattering spectra only appeared at the polarization angles where PL splitting was observed. To exclude the possibility that the new scattering peaks are PL signals excited by the halogen lamp in the dark-field scattering measurements, we performed a control experiment by introducing a 730–760 nm bandpass filter right after the halogen lamp in the setup (Figure S7a). In this way we filtered out most optical energy that could potentially contribute to PL excitation but retained the energy necessary for exciting scattering at those wavelengths. Despite the introduction of the filter, the scattering-peak-splitting features remained (Figure S7b). These features are therefore likely the result of altered scattering signals owing to strong plasmon–exciton coupling.

The emission-polarization-dependent PL peak splitting appears in many other samples at arbitrarily determined excitation polarization angles (Figures S8 and S9). Such features, however, were hardly found in the scattering and PL spectra without detection polarization resolution (Figures S5 and S9). Additional experiments further revealed that most samples exhibiting the PL and scattering peak splitting at certain detection polarization angles also display a weak polarization dependence for the scattering intensity under the excitation of unpolarized white light. We performed SEM and atomic force microscopy (AFM) imaging on one of these samples. The ND exhibits a rather circular geometry under SEM imaging (Figure S9d). However, AFM imaging revealed slight ∼5° tilting for the ND. According to the SEM images of the evaporated Au films used for the device fabrication (Figure S9d), the evaporated Au films have Au grains with varying sizes, morphologies, and orientations on the top surface. Such Au grains potentially induce slight tilting for deposited Au NDs and break the rotational symmetry of the resultant structures, resulting in the polarization-dependent scattering of the nanocavity. The nanocavities thus exhibit an anisotropic polarization-dependent plasmon resonance property. Bright A excitons with varying in-plane dipole orientations interact with cavity plasmons at different coupling strengths. We therefore observed variations in the PL spectra at different emission polarization angles. A similar particle-tilting-induced anisotropic polarization effect has been reported by another study involving plasmonic nanoparticle-on-mirror cavities.^40^

One can utilize the polarization anisotropy in the scattering as an indicator to screen nanocavities that enable strong plasmon–exciton coupling, since the relative orientations and strengths of the emitter dipole and the cavity mode are critical for their strong coupling.^41^ The PL and scattering spectra of the filtered nanocavities that couple to TMDC bright A excitons exhibit consistently the double-peaked spectral characteristics at certain detection polarization angles (Figure 2d, pink shaded spectra). Slightly shifting the toroidal plasmon mode by varying the ND diameter produced different relative PL peak heights. Electromagnetic field simulations indicate that the charge distributions of the toroidal mode dominate within −100 to +100 nm relative to the first-order toroidal mode peak wavelength (Figure S10).^32^ As a result, for the top first and second spectra in Figure 2d, although the toroidal resonance peak wavelength is approximately 70–50 nm shorter than the bright A exciton PL wavelength, bright A excitons interact with the tail of the first-order toroidal resonance. Strong plasmon–exciton coupling was confirmed by an anticrossing behavior between the plasmonic and excitonic branches in the PL spectra with a 21.28 ± 1.94 meV Rabi splitting at zero detuning (Figure 2e). A coupling strength *g* of 10.64 ± 0.97 meV was determined according to a two-coupled-harmonic-oscillator model (Supplementary Note 1), which meets the strong coupling criterion 2*g* > (γ_pl_–γ_ex_)/2,^42–44^ where *g*, γ_pl_, and γ_ex_ are the coupling strength, plasmon line width, and bright A exciton line width, respectively, but does not fulfill the stricter criterion 2*g* > (γ_pl_ + γ_ex_)/2.^45^ In this study, the plasmon and bright A exciton line widths were extracted to be 82.1 ± 11.0 and 51.6 ± 0.7 meV from the experimental spectra through Lorentz and Gaussian fittings, respectively. Fulfilling the first criterion guarantees two real solutions in the coupled-oscillator model when the emitter is resonantly tuned to the plasmon resonance and can be interpreted as the definition of the lower bound for the strong-coupling regime.^45^ However, it does not ensure clear separate peaks in the measured spectra. Therefore, the latter criterion as a stricter one is often introduced. In our case, splitting is indeed present in both PL and scattering spectra. As a result, we can safely state that the (WSe_2_ monolayer)–(NDoM cavity) measured here were found to be at the onset of the strong-coupling regime. When the toroidal resonance was shifted to the dark A exciton energy, the characteristic peak splitting disappeared at all detection polarization angles. Instead, bright and spin-forbidden dark A exciton emissions appeared at 1.65 and 1.60 eV in the PL spectra, respectively (Figure 2d).^46^

### Room-Temperature Valley Coherence

We next studied the room-temperature valley coherent properties of the plasmon–exciton polaritons. We constructed NDoM cavities with the toroidal plasmon resonance aligned with the WSe_2_ bright A exciton emission energy (Figure S11) and collected emission-polarization-resolved PL spectra at varied excitation polarization directions. Figure 3a shows the spectra in the case that the input and output have co- and cross-linear polarization for three different input linear polarization orientations. In each panel of Figure 3b, the integrated PL intensity, as a function of polarizer orientation θ, is fitted to *A*sin^2^(θ + ϕ) + *C*_0_. The agreement between excitation and emission polarization was found to be preserved irrespective of the scattering polarization of the nanocavity to unpolarized white light. As long as the scattering polarization anisotropy was kept at a small value, the PL polarization modulation by the plasmonic nanoantenna effect would not dominate. The colinearly polarized PL emissions with the excitation light confirm the room-temperature intervalley phase coherence between the valley polaritons/excitons before they decay to photons. We employed the degree of PL linear polarization (ρ_l_) to quantify the degree of valley coherence, which is expressed as^17^

$$\rho_{l}=\frac{I_{\mathrm{co}}-I_{\mathrm{cross}}}{I_{\mathrm{co}}+I_{\mathrm{cross}}}$$ 1

where *I* is the integrated PL intensity and “co” (“cross”) indicates that the input and output linear polarizers are parallel (perpendicular). ρ_l_ of the WSe_2_-monolayer-sandwiched NDoM cavity in Figure 3 was determined to be 16.6 ± 2.3, 7.6 ± 6.3, and 10.8 ± 2.3% at an excitation polarization angle of 120°, 90°, and 60°, respectively, with an average of 11.6 ± 2.4%, which is comparable to the values achieved with a WSe_2_ monolayer embedded in a DBR cavity at room temperature (Table S1).

**Figure 3:**
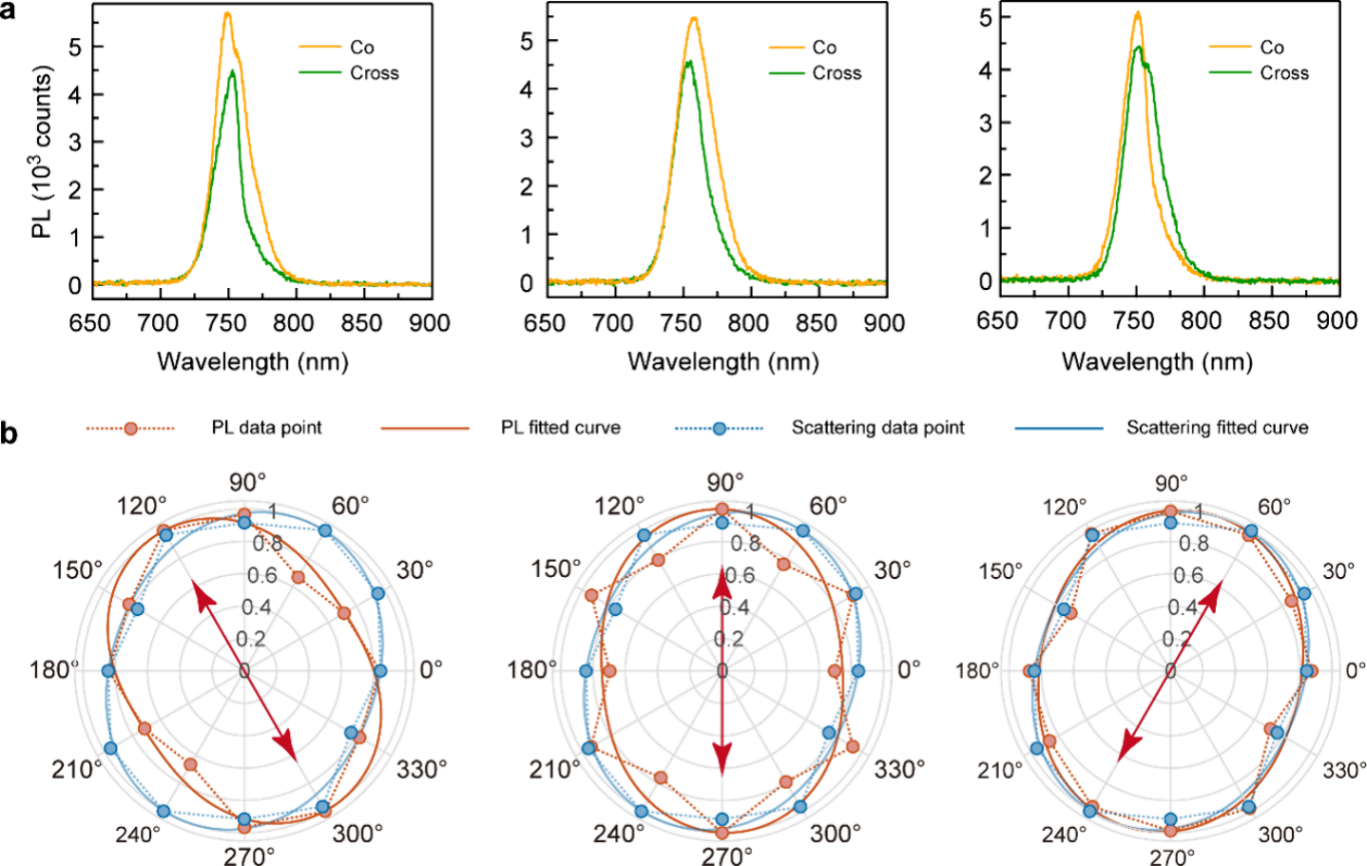
Observation of PL valley coherence in a WSe_2_-monolayer-sandwiched NDoM nanocavity. (a) PL spectra of the nanocavity with the toroidal plasmon resonance aligned with the WSe_2_ bright A exciton emission energy when excitation and detection polarizations are aligned (co-) and perpendicular (cross-) at varying excitation (input) polarization directions. The excitation polarization angles from left to right are 120°, 90°, 60°, respectively. (b) Integrated PL intensity as a function of the emission polarization angle for the given linearly polarized excitation directions (red arrow). The solid lines are fittings according to *I* = *A*sin^2^(θ + ϕ) + *C*_0_ to extract emission polarization angles. The dashed lines and solid circles depict the integrated intensities derived from the measured spectra.

The linearly polarized component of the nanocavity PL signal along the excitation polarization direction shown in Figure 3 is a consequence of coherent superposition of the K and K′ polaritons or excitons in the plasmonic cavity. To examine the capability of preserving valley information by the plasmonic nanocavity, we performed helicity-resolved PL spectroscopy measurements on the same NDoM cavity depicted in Figure 3 by subjecting it to σ– and σ+ laser excitation. The valley contrast is quantified by the degree of valley polarization (ρ_c_)­

$$\rho_{c}=\frac{I_{\sigma +}-I_{\sigma -}}{I_{\sigma +}+I_{\sigma -}}$$ 2

where *I*_σ+_ and *I*_σ–_ represent the helicity-resolved PL intensities. The NDoM cavity shows valley polarization of ρ_c_ = −15.0 and +30.6% for σ– and σ+ excitation (Figure 4a), respectively. The opposite values of ρ suggest that the polarization of the excited valley (K′ or K) is kept and detectable at room temperature through coupling to the toroidal resonance of the NDoM cavity, even though a large portion of valley information still gets lost mainly through strong intervalley scattering at elevated temperatures. Since our Au NDs exhibit a certain degree of deviation from the perfect circular shape (Figures 2a and S9) and experience slight tilting along random axes, the larger valley splitting at the σ+ excitation can be attributed to the chiral Purcell effect caused by the ND-tilting-induced asymmetry for σ+ and σ– local density of states (Figure S12), which is similar to the case of a previous work.^40^ While chiral nanocavities enable selective depopulation of valley-polarized excitons through the chiral Purcell effect, they affect the preservation of valley coherence and are intentionally avoided in this work. For bare WSe_2_ monolayers at the same temperature, we measured ρ_c_ = −0.01 and −0.02% under σ– and σ+ laser excitation (Figure 4b), indicating that valley polarization completely vanished without the plasmonic cavity.

**Figure 4:**
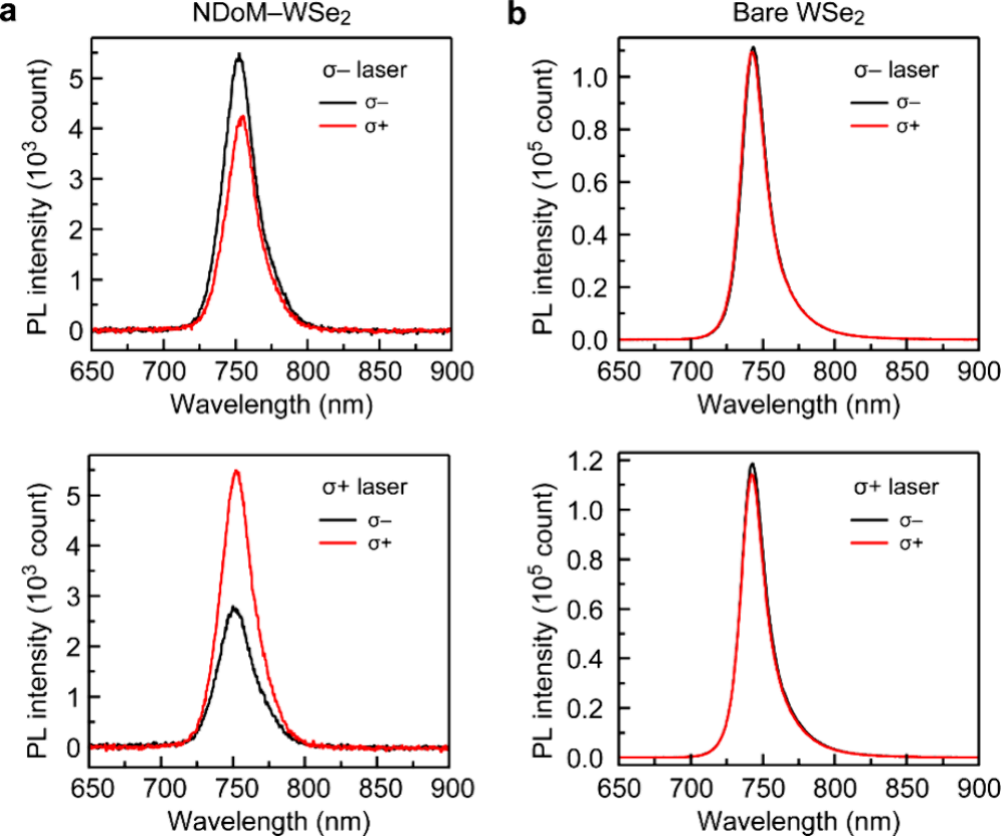
Valley-polarized emissions of a NDoM-cavity-coupled WSe_2_ monolayer in comparison with a bare WSe_2_ monolayer. (a) Circularly polarized PL spectra of the nanocavity shown in Figure 3 pumped with σ– (upper) and σ+ (lower) laser beams. (b) Circularly polarized PL spectra of a bare WSe_2_ monolayer on a polydimethylsiloxane (PDMS) substrate. The measurements were conducted at room temperature.

### Electromagnetic Simulations

To gain insight into the interactions between the toroidal plasmon resonance and TMDC bright A excitons, we carried out electromagnetic simulations on the WSe_2_-monolayer-sandwiched NDoM cavity with a disk diameter of 103 nm for both in-plane and out-of-plane excitation using the finite-difference time-domain (FDTD) method. We modeled the NDoM cavity based on the experimentally obtained structural parameters and varied the thickness of the Al_2_O_3_ layer to investigate the impact of the gap thickness on the resonance wavelength. The calculated scattering spectra under both in-plane and out-of-plane excitation demonstrate a deviation in energy and a dissimilar relative peak intensity compared to the corresponding measured result (Figure S13). By tilting the ND against the *y* axis (Figure S14a), the differences in energy and relative peak heights between the simulated and measured spectra became smaller (Figure S14b). The simulated scattering spectrum appears in a shape with a prominent scattering peak at a longer wavelength and bumps at shorter wavelengths, which resembles the measured spectrum. The simulation results support our preceding hypothesis that the ND is slightly tilted on the substrate.

We compared the electromagnetic near-field properties of the NDoM toroidal plasmon resonance with and without ND tilting. In the absence of ND tilting, a toroidal plasmon mode with its resonance wavelength λ_T_ = 739 nm can only be excited by an out-of-plane polarized light (Figure 5a–e),^32^ exhibiting magnetic field confinement in a toroidal configuration (Figure 5b). The magnetic field vectors within the gap behave in circular trajectories parallel to the ND surface (Figure 5c), indicating the orientation of the induced toroidal moment. A significantly enhanced electric field is generated at the center of the cavity gap (Figure 5d), which possesses a field configuration similar to that of a skyrmion (Figure 5e,f).^47^ The tilting of the Au ND eliminates the requirement that an out-of-plane polarized light should be applied to excite the toroidal resonance. Instead, the toroidal resonance can be effectively excited at almost the same wavelength (λ_T_ = 747 nm) by in-plane polarized light with a polarization direction perpendicular to the tilting axis (Figure 5g–i). Excitation under an in-plane polarized light with a polarization direction parallel to the tilting axis would generate an in-plane magnetic mode at a shorter wavelength,^32^ which exhibits a strong scattering intensity, a weaker electric field enhancement and a more prominent magnetic field enhancement (Figure S15). The presence of a circular magnetic field configuration was observed near the closest point between the tilted Au ND and the Au film (Figure 5h–j), implying the preservation of the topological electric field texture. Most importantly, the optical electric field inside the nanocavity gets highly confined, with the maximal enhancement factor further increased because of ND tilting. Both the vertical and horizontal components of the optical electric field inside the nanocavity are large, enabling the strong interaction of the toroidal plasmon mode with both in-plane polarized bright A excitons and out-of-plane polarized dark A excitons of the WSe_2_ monolayer in our experiments. Quantitative analysis of the field data revealed that the electric field configuration of the toroidal resonance exhibits a topological texture. The topological charge *Q* is defined as

$$Q=\frac{1}{4\pi }\int N\cdot \left(\frac{\partial N}{\partial x}\times \frac{\partial N}{\partial y}\right)dxdy$$ 3

where ***N*** represents the electric field unit vector. *Q* has its value very close to unity, confirming the skyrmion nature of the optical field. For a NDoM cavity constructed out of an untitled Au ND, the calculated topological charge *Q* for the electric field configuration remains consistently at 0.98 across all phases (Figure S16), indicating both mode purity and the skyrmion nature of the toroidal mode. However, as the ND is tilted away from the horizontal alignment to a small angle (5°), the mode purity is damaged. Other modes such as the in-plane magnetic mode (Figure S15) appear at a similar energy and contribute to field interference and the overall electric field configuration. The topological charge of a NDoM cavity constructed out of a tilted ND was found to vary with phase, reaching a value of 1 at a specific phase only (Figure S16). Such topological stability of the optical field enabled by the toroidal plasmon resonance agrees well with the properties of skyrmions (*Q* = ±1) observed in other physical systems and should benefit applications in information storage and processing. Moreover, the skyrmion nature ensures that the optical electric field points toward the entire 4π solid angle inside the nanocavity, allowing the spatial-perturbation-insensitive efficient coupling between the plasmon resonance and different TMDC excitons with varying emission dipole orientations. Our plasmonic NDoM structures therefore offer a promising robust platform for the modulation of various excitons inside 2D TMDC materials.

**Figure 5:**
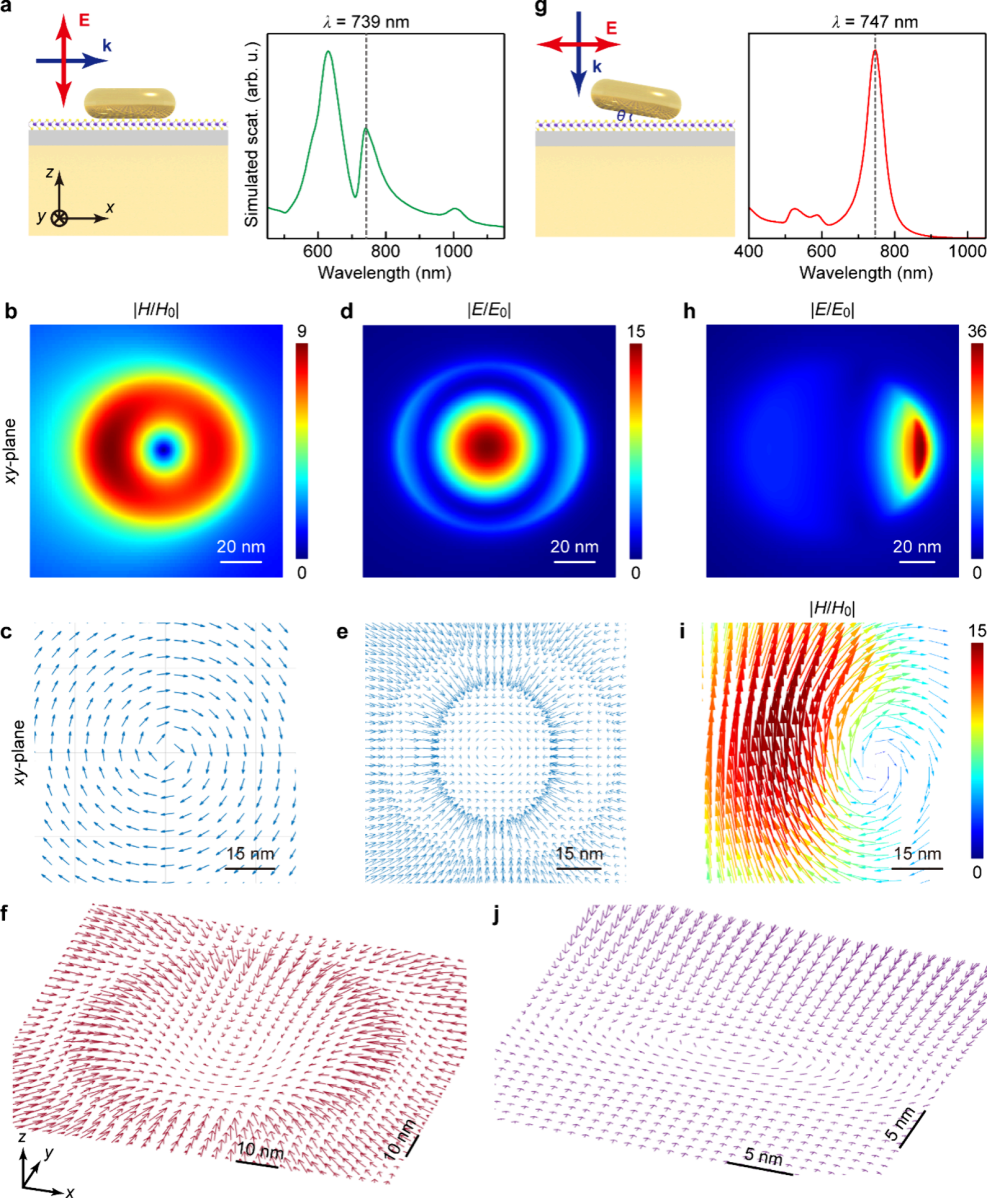
Toroidal plasmon resonance in the NDoM structure. (a) Schematic of the simulation model for the NDoM structure excited by out-of-plane polarized light (left) and simulated scattering spectrum (right). The gray dashed line indicates the generation of the toroidal resonance at 739 nm. (b, c) Simulated magnetic field enhancement contour and normalized field vectors at the toroidal resonance, respectively. (d, e) Simulated electric field enhancement contour and normalized field vectors in the nanocavity at the toroidal resonance, respectively. (f) Stereoscopic view of the electric field vectors of (d). (g) Schematic of the simulation model for the NDoM structure with a tilted Au ND excited by in-plane polarized light (left) and simulated scattering spectrum (right). The gray dashed line indicates the generation of the toroidal resonance at 747 nm. The tilting angle θ of the Au ND is ∼5° and the Al_2_O_3_ layer thickness is 2 nm, in agreement with the experimental conditions. (h, i) Simulated electric field enhancement contour and magnetic field vectors in the tilted nanocavity at the toroidal resonance, respectively. (j) Stereoscopic view of the electric field vectors of (h). All the field distributions were obtained at the *xy* plane located at the center of the WSe_2_ monolayer.

We should point out that in our experiments the colinearly polarized PL emissions with the excitation light, which indicates room-temperature valley coherence, can be observed even at detection polarization angles where a distinct PL peak splitting that reflects the toroidal plasmon–exciton interaction entering the strong coupling regime is absent. One therefore cannot ascertain that the optically accessible room-temperature valley coherence or valley polarization is dominantly originated from the strong-coupling-formed plasmon–exciton polariton by excluding the possibility of the near-field-enhanced radiative decay of valley excitons. It is more likely that both mechanisms exist in our plasmonic NDoM cavities. The simulated Purcell factor of our system for an in-plane transition dipole has a moderate value of 559. While exciton radiation in TMDC monolayers has a lifetime of hundreds of picoseconds, valley lifetime is only hundreds of femtoseconds to 1 ps.^48^ Through the Purcell effect, our NDoM cavities barely meet the level where valley information can be retained in the exciton PL.

In the picture of strong plasmon–exciton coupling, the decay mechanism of the strong-coupling-formed polaritons in the NDoM cavity can be interpreted with the valley-specific Jaynes-Cummings model.^17^ The model consists of two valley-resolved exciton levels (K and K′ valleys) and two helicity-resolved cavity plasmon modes (RCP and LCP cavities). Coupling between the excitons and plasmons results in coherent transitions between the K/K′ excitonic energy states and the RCP/LCP cavity plasmon energy states. The four states are connected to the ground state by their individual population decay. The laser pumping only excites the two valley excitonic states. In the strong coupling regime, the polaritonic degree of valley coherence and valley polarization can be expressed in terms of the following rates^17^

$$\rho_{l}=\frac{1}{1+\frac{\gamma_{v}+\gamma_{\mathrm{dep}}}{\gamma_{c}+\gamma_{\mathrm{ex}}}},\;\rho_{c}=\frac{1}{1+\frac{2\gamma_{v}}{\gamma_{c}+\gamma_{\mathrm{ex}}}}$$ 4

where γ_c_, γ_ex_, γ_v_, and γ_dep_ represent the cavity photon relaxation, exciton relaxation, intervalley relaxation, and pure dephasing rates. However, the exciton valley coherence or valley polarization helicity, i.e., ρ_l_ or ρ_c_, of a bare monolayer WSe_2_ does not include the cavity photon relaxation term γ_c_. The incoherent intervalley scattering (γ_v_) leads to significant valley decoherence and depolarization, while the pure dephasing process (γ_dep_) results in valley decoherence. By coupling to the near-field of the toroidal plasmon resonance, exciton polaritons obtain an extra population relaxation path (γ_c_), which is free of decoherence or depolarization because the toroidal mode is immune to any intervalley scattering or exciton dephasing processes. Therefore, valley coherence and polarization contrast of the polaritons can be substantially improved by both the accelerated relaxation process and the suppressed decoherence/depolarization means. In the picture of plasmon-enhanced valley exciton radiative decay without entering the strong coupling regime, the degree of valley coherence and valley polarization, ρ_l_ and ρ_c_, become^49,50^

$$\rho_{l}=\frac{1}{1+\frac{\gamma_{v}+\gamma_{\mathrm{dep}}}{\gamma_{\mathrm{ex}}}},\;\rho_{c}=\frac{1}{1+\frac{2\gamma_{v}}{\gamma_{\mathrm{ex}}}}$$ 5

with the expression the same as those of the bare monolayer WSe_2_. The excitation of the cavity plasmon resonance greatly enlarges the local density of optical states, and as a result largely enhances the radiative decay rate of the excitons of the TMDC monolayer inside the nanocavity, giving rise to a remarkably increased exciton recombination rate γ_ex_ in eq 5. The accelerated radiative decay process alone can lead to the optical readout of the valley coherence and polarization contrast information on bright WSe_2_ A excitons at room temperature. Despite the debate above, we still believe that the strong coupling between the cavity plasmon and WSe_2_ monolayer bright A exciton is critical for the room-temperature valley coherence observed in our PL experiments, since we could measure colinearly polarized PL emissions with the excitation light only in samples that exhibited the PL peak splitting in certain emission polarization angles. Further investigations should be performed, for example, by employing time- and polarization-resolved PL spectroscopy, to reveal the detailed mechanism.

## Conclusions

In conclusion, our work presents evidence for the strong coupling and valley coherence of individual WSe_2_-monolayer-sandwiched NDoM structures at room temperature. The toroidal resonance generated by the NDoM cavity exhibits topological electric field nanotexture of a skyrmion and gives rise to electric field enhancement |*E*/*E*_0_| up to ∼36 in the gap region. Owing to a slight tilting of the Au ND, the requirement for an out-of-plane electric field to excite the toroidal resonance is eliminated. We have observed polarization-dependent strong coupling between bright A excitons and the toroidal plasmon mode characterized by a Rabi splitting of 21.3 ± 1.9 meV in the PL spectra. The strong plasmon–exciton coupling enhances the valley coherence substantially, resulting in linearly polarized PL emissions with an average linear polarization degree of 11.6 ± 2.4%. Our study demonstrates the preservation of valley coherence at room temperature using plasmonic nanocavities scaled down to the single-nanoparticle level, which potentially provides an ultracompact platform for the development of valleytronic devices and brings us closer to meeting the demands of quantum optics applications. In addition, nanoparticle-on-mirror structures have enabled intriguing investigations into the nonlinear optical properties of TMDC monolayers.^39,51,52^ To leverage the valley degree of freedom, future effort can be made to explore valley-coherent nonlinear processes, where the valley polarization is manipulated or generated through nonlinear interactions, enhanced by the near-fields of specifically designed nanoparticle-on-mirror systems.

## Methods

### Sample Preparation

Au substrates were prepared by depositing a 5 nm-thick Ti adhesion layer on a Si wafer through electron-beam evaporation, followed by the deposition of a 100 nm-thick Au film. An Al_2_O_3_ layer was then deposited on the Au film through ALD. To characterize the actual thickness of the Al_2_O_3_ layer, chemically synthesized Au microplates were drop-cast onto a TEM grid, which was placed into the ALD chamber together with the Au film for Al_2_O_3_ deposition. The thickness of the Al_2_O_3_ layer was determined to be 2.4 nm by measuring the edges of the Au microplates through high-resolution TEM after deposition.^32^ WSe_2_ monolayers were exfoliated from bulk WSe_2_ and dry-transferred onto the Au/Al_2_O_3_ substrates with a home-built transfer system. After the preparation of the Au/Al_2_O_3_/WSe_2_-monolayer structures, Au NDs that were predeposited on a PDMS film were dry-transferred from the PDMS film to the top of the WSe_2_ monolayer to construct WSe_2_-monolayer-sandwiched NDoM structures. The prepared structures were then immersed in ethanol for 30 s to remove the residual cetyltrimethylammonium bromide (CTAB) molecules at the surface of the chemically synthesized NDs. To eliminate the influence of PL signals from the WSe_2_ monolayer off the cavities, we deposited monodisperse ultrasmall Au nanoparticles with an average diameter of 3 nm on the hybrid structures through electron-beam evaporation to quench the PL signals of exposed WSe_2_.

### Dark-Field Scattering and PL Spectroscopy

The optical properties of individual plasmonic nanostructures were investigated with the aid of a home-built single-particle dark-field scattering imaging and spectroscopy system. The measurements were carried out using an upright optical microscope (Olympus BX60) equipped with a quartz-tungsten-halogen lamp (100 W), a monochromator (Princeton Instruments, SP2300i), and a charge-coupled device camera (Princeton Instruments Pixis 400, cooled to −70 °C). A 100× dark-field objective (numerical aperture 0.9) was employed to create an inverted hollow light cone illumination for the excitation of individual nanostructures and the collection of the scattered light. PL measurements were conducted on the same optical microscope with an excitation wavelength of 633 nm. The laser was directed into the optical microscope and focused by a 100× objective. A long-pass filter was used to block the reflected and scattered laser light and transmit the PL signal to the camera or the spectrometer. PL spectra were measured by focusing the excitation laser beam onto individual nanostructures and collecting the PL signal from the laser focus. Linear-polarization-resolved scattering and PL spectra were acquired by placing a variable linear polarizer in front of the spectrometer. Circularly polarized laser light was generated by first transmitting the laser through a linear polarizer and then a quarter-wave plate. The angle between the linear polarization axis and the fast axis of the quarter-wave plate was set at 45° or −45°. The helicity-resolved PL spectra were acquired by first transmitting the PL signal through a quarter-wave plate and then a linear polarizer.

### FDTD Simulations

FDTD simulations were performed using FDTD Solutions 8.19 developed by Lumerical Solution. The Au ND was modeled as a cylinder surrounded by a concentric torus. The thickness and radius of the cylinder were set at 52 and 43.5 nm, respectively. The inner and outer radii of the torus were set at 8 and 43.5 nm, respectively. The NDoM model was constructed by a circular Au nanodisk, a 1 nm-thick WSe_2_ layer, an Al_2_O_3_ layer with varying thickness *d* and a 100 nm-thick Au film. To account for the presence of residual CTAB molecules on the surface of the chemically synthesized ND, we introduced a 1 nm gap between the ND and the WSe_2_ layer whose refractive index was assigned to be 1.0. The in-plane permittivity was taken from a previous work^53^ and the out-of-plane permittivity was set at 7.4 for the real part,^54^ with the imaginary part of the out-of-plane permittivity ignored. The refractive indexes of Al_2_O_3_ and air were set at 1.4 and 1.0, respectively. The dielectric function of Au was taken from Johnson and Christy’s data. The mesh sizes of 0.5 and 1.0 nm were employed in the gap and the other parts of the structure, respectively. The total-field scattering-field source module was launched for the simulation of the scattering spectra and field enhancement contours.

## Supplementary Material



## Notes

Circular Au ND samples; exfoliated WSe_2_ monolayer sandwiched between circular Au NDs and a Au film; ultrasmall Au nanoparticles; scattering and PL spectra of NDoM structures; emission-polarization-resolved spectral measurements; two-coupled-harmonic-oscillator model; FDTD-simulated spectra, electric field distributions, and charge distributions; and topological charge calculations (PDF)
