# Supplementary material for: Coherent Polaritons in WSe2‑Monolayer-Sandwiched Au-Nanodisk-on-Mirror Structures
Source: ACS Nano. 2025 Jul 4;19(27):25284–94. doi: 10.1021/acsnano.5c06093 (PMC12269354; doi:10.1021/acsnano.5c06093)
Supplement: Supplementary file 1 [file nn5c06093_si_001.pdf]

Supporting Information

Coherent Polaritons in WSe_2-monolayer-sandwiched Au-nanodisk-on-mirror Structures

He Huang,[1] Bo Tian,[2] Yang Chen,[1] Xinyue Xia,[1] Ximin Cui,[3] Lei Shao,[2,] Huanjun Chen,[2] and Jianfang Wang[1,]**

[1]Department of Physics, The Chinese University of Hong Kong, Shatin, Hong Kong SAR 999077, China.

[2]State Key Laboratory of Optoelectronic Materials and Technologies, Guangdong Provincial Key Laboratory of Display Materials and Technologies, School of Electronics and Information Technology, Sun Yat-sen University, Guangzhou, Guangdong 510275, China.

[3]State Key Laboratory of Radio Frequency Heterogeneous Integration, College of Electronics and Information Engineering, Shenzhen University, Shenzhen, Guangdong 518060, China.

*Email: shaolei5@mail.sysu.edu.cn (L.S.); jfwang@phy.cuhk.edu.hk (J.F.W.)

Content

Figure S1: Circular Au NDs.

Figure S2: AFM image and height profile of a piece of mechanically exfoliated WSe_2 monolayer sandwiched between circular Au NDs and a Au film.

Figure S3: Ultrasmall Au nanoparticles.

Figure S4: Effect of the deposition of ultrasmall Au nanoparticles.

Figure S5: Scattering and PL spectra of NDoM structures.

Figure S6: Emission-polarization-resolved spectral measurements.

Figure S7: Scattering spectra acquired using a halogen lamp with and without a 730–760 nm bandpass filter.

Figure S8: WSe_2-monolayer-sandwhiched NDoM cavities showing the polarization-dependent PL splitting.

Figure S9: Optical and structural characterization of a typical WSe_2-monolayer sandwiched NDoM structure.

Figure S10: Charge distribution and magnetic vector field map of the toroidal mode within −100 nm to +100 nm relative to the peak wavelength.

Supplementary Note 1: Two-coupled-harmonic-oscillator model.

Figure S11: Scattering and PL measurements of a NDoM cavity with the toroidal plasmon resonance aligned with the WSe_2 A exciton emission energy.

Table S1. Valley coherence performance reported in other literature.

Figure S12: Chiral Purcell effect.

Figure S13: FDTD simulation of the scattering spectra of a WSe_2-monolayer-sandwiched NDoM cavity under in-plane and out-of-plane excitations.

Figure S14: Effect of ND tilting on the scattering spectrum.

Figure S15: In-plane magnetic mode.

Figure S16: In-plane magnetic mode. Calculated topological charges as a function of the electric field phase for NDoM cavities with an untitled Au ND and a Au ND tilted by 5°.

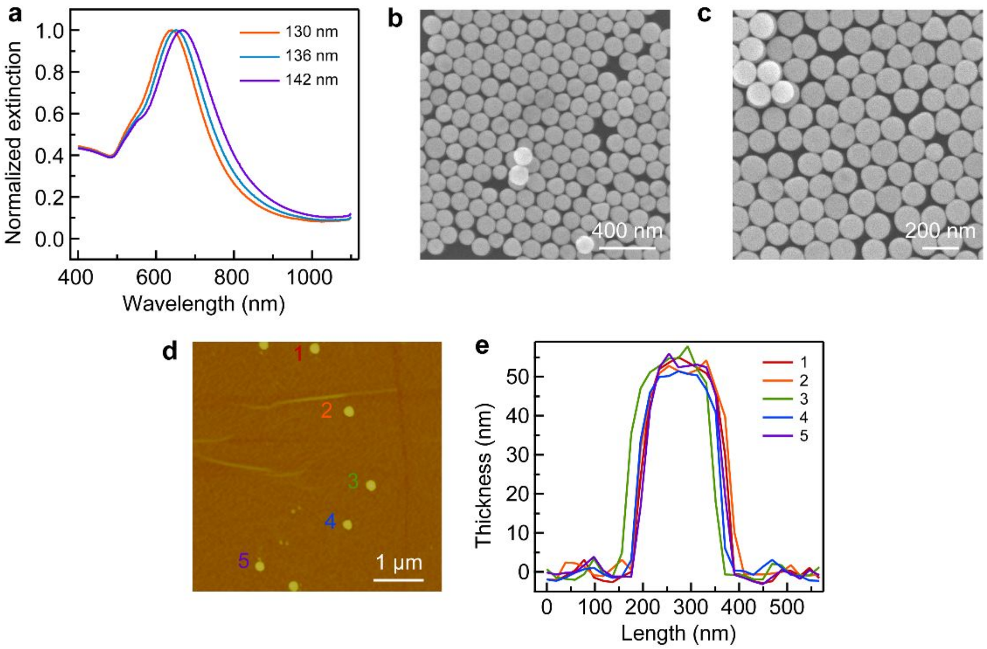

Figure S1. Circular Au NDs. (a) Measured extinction spectra of the ND samples dispersed in water with three different average diameters, 130 ± 8 nm, 136 ± 4 nm, and 142 ± 2 nm, respectively. (b,c) SEM images of the circular ND samples with average diameters of 136 ± 4 nm and 142 ± 2 nm. (d) Atomic force microscopy (AFM) height image of five WSe_2-monolayer-sandwiched NDoM cavities. (e) Height profiles of the five NDs in (d). The average thickness of the NDs is 53 ± 1 nm.

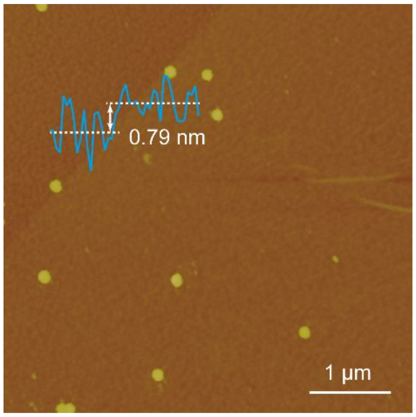

Figure S2. AFM characterization of WSe_2 monolayers. Shown are the AFM image and height profile of a piece of mechanically exfoliated WSe_2 monolayer sandwiched between circular Au NDs and a Au film.

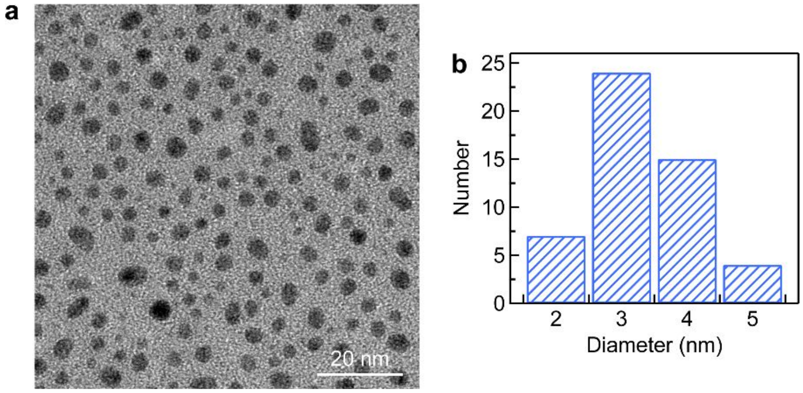

Figure S3. Ultrasmall Au nanoparticles. (a) TEM image. The sample was prepared by putting a TEM grid into the chamber together with the NDoM devices for Au nanoparticle deposition through electron beam evaporation. (b) Histogram of the nanoparticle diameter obtained from (a). The average diameter of the Au nanoparticles was measured to be 3.4 ± 0.7 nm.

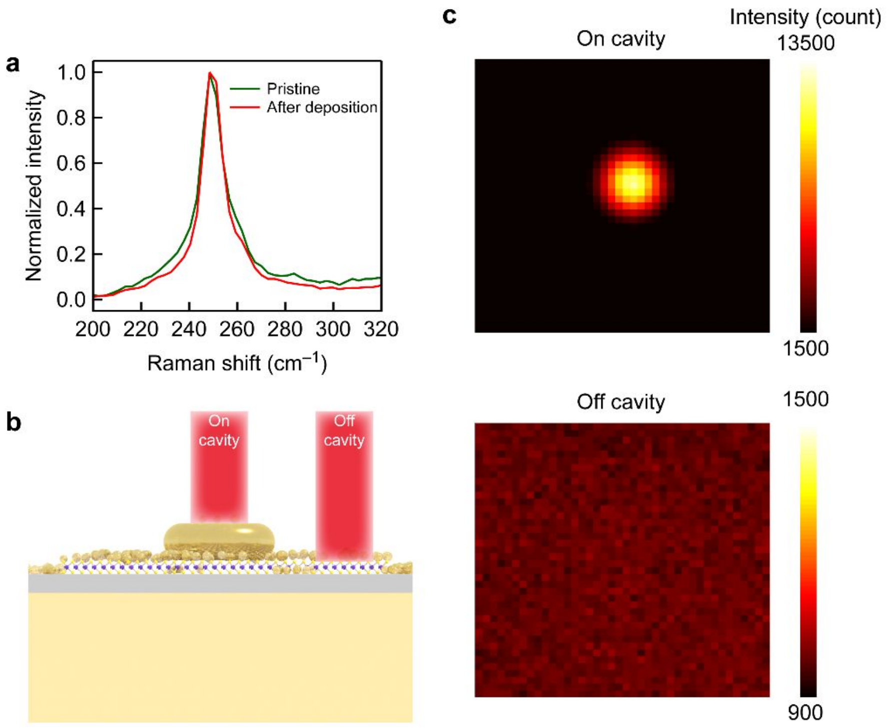

Figure S4. Effect of the deposition of ultrasmall Au nanoparticles. (a) Raman spectra of a WSe_2 monolayer before and after the deposition of ultrasmall Au nanoparticles on the top. (b) Schematic illustrating on-cavity and off-cavity probing. (c) PL images at on-cavity probing (top) and off-cavity probing (bottom). The PL of the WSe_2 monolayer off the cavity is quenched by the ultrasmall Au nanoparticles.

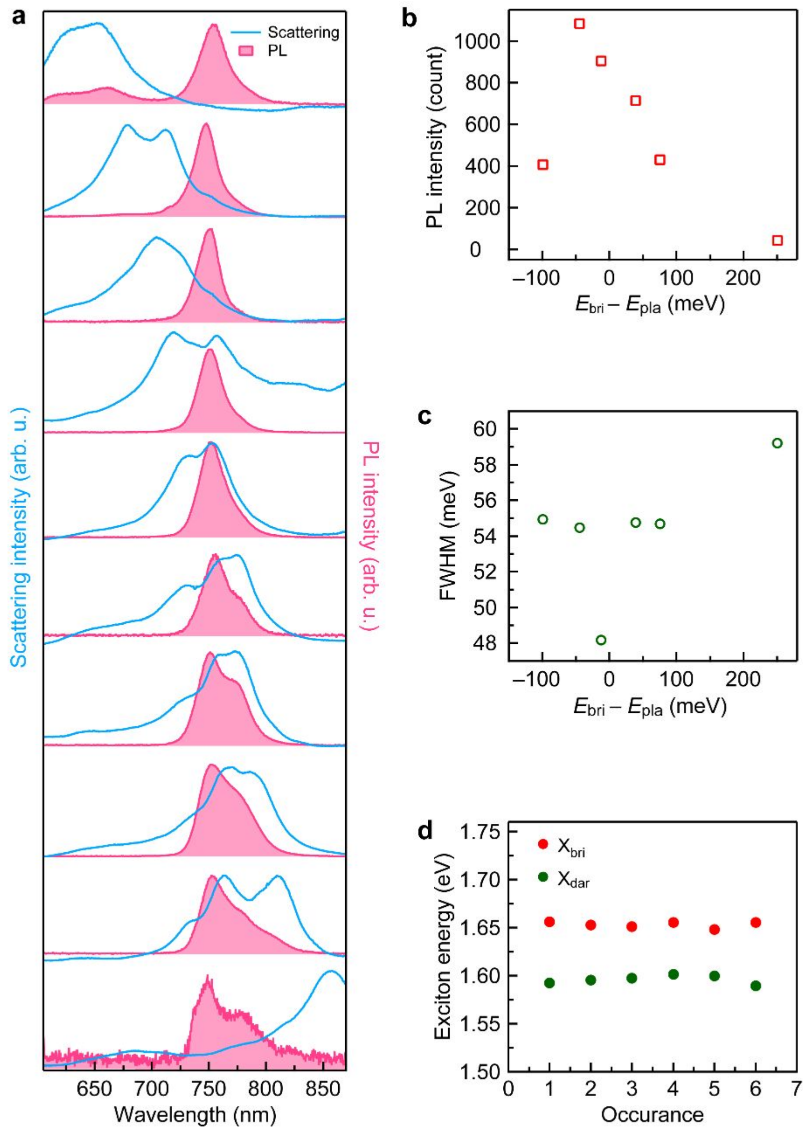

Figure S5. Scattering and PL spectra of the NDoM structures. (a) PL and scattering spectra of the NDoM structures with different toroidal resonance energies. (b,c) PL intensity and full width at half maximum (FWHM) of bright A excitons extracted through Lorenz fitting as a function of the energy difference between the bright A exciton and the toroidal resonance. The PL intensities were obtained by integrating the PL peaks of bright A excitons. (d) Statistical analysis of the bright A exciton (X_{bri}) and dark A exciton (X_{dar}) energies observed in the PL spectra in (a).

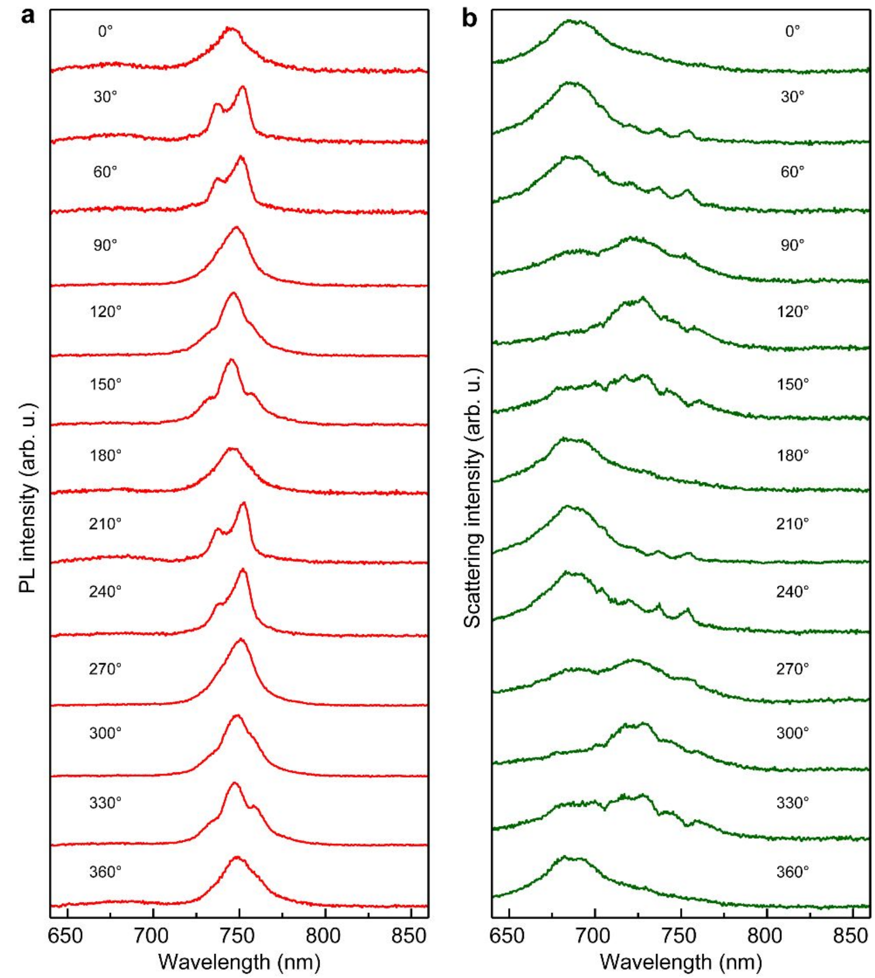

Figure S6. Emission-polarization-resolved spectral measurements. (a,b) Polarization-resolved PL (left) and scattering spectra (right) of a typical NDoM cavity. The detection polarization angle is indicated next to each spectrum.

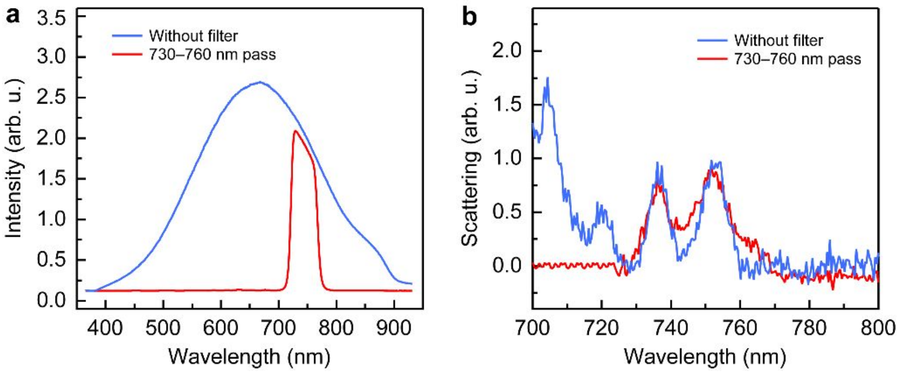

Figure S7. Scattering spectra acquired using a halogen lamp with and without a 730–760 nm bandpass filter. (a) Spectra of a halogen lamp before and after transmitting through a 730–760 nm bandpass filter. (b) Scattering spectra of the NDoM cavity in Figure S6 under the excitation of the halogen lamp with and without the filter.

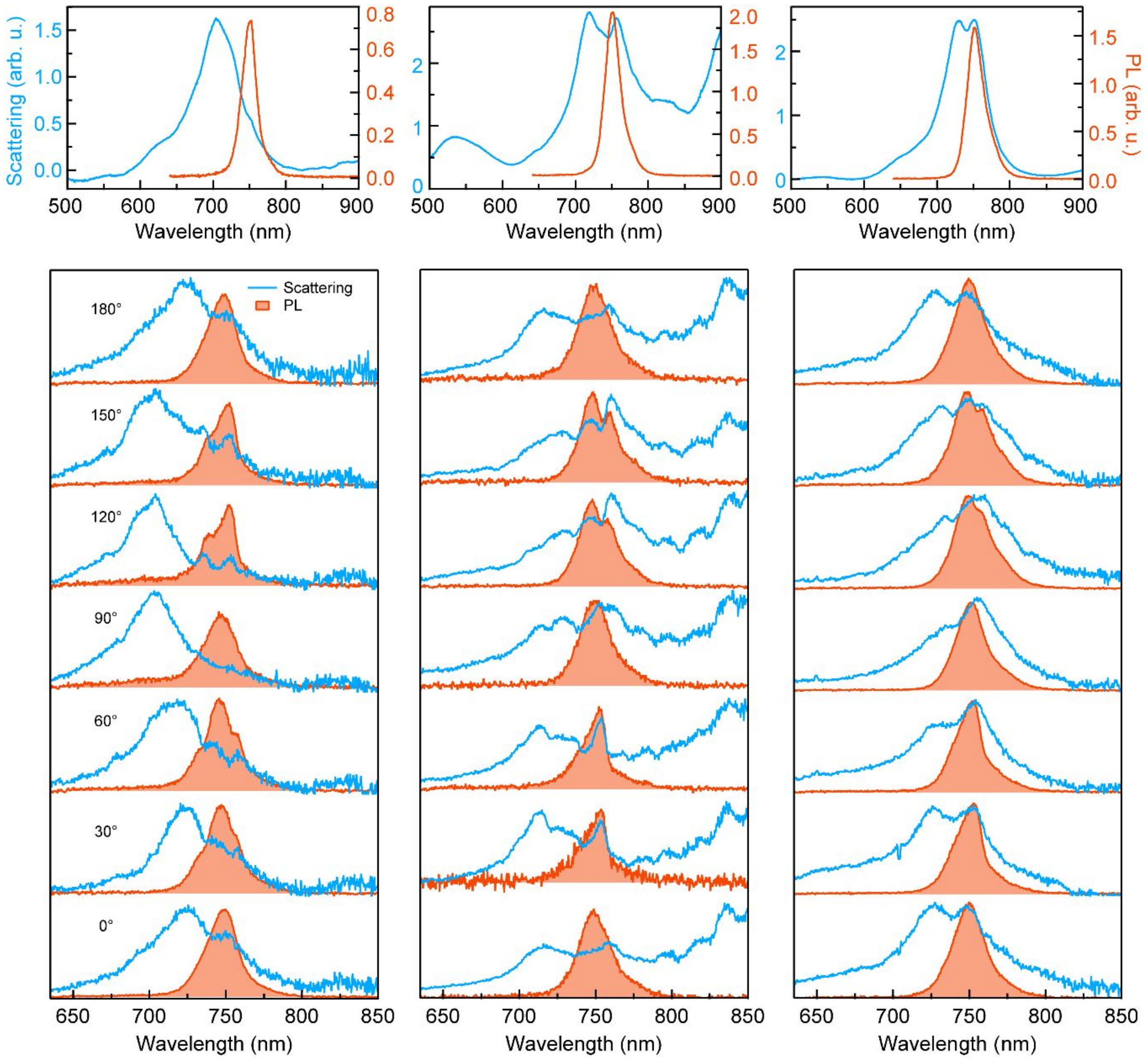

Figure S8. WSe_2-monolayer-sandwhiched NDoM cavities showing the polarization-dependent PL splitting. The upper panel shows the scattering and PL spectra of three different NDoM cavities without polarization resolution. The lower panel shows their corresponding polarization-resolved scattering and PL spectra. The detection polarization angles are indicated in the corresponding plots.

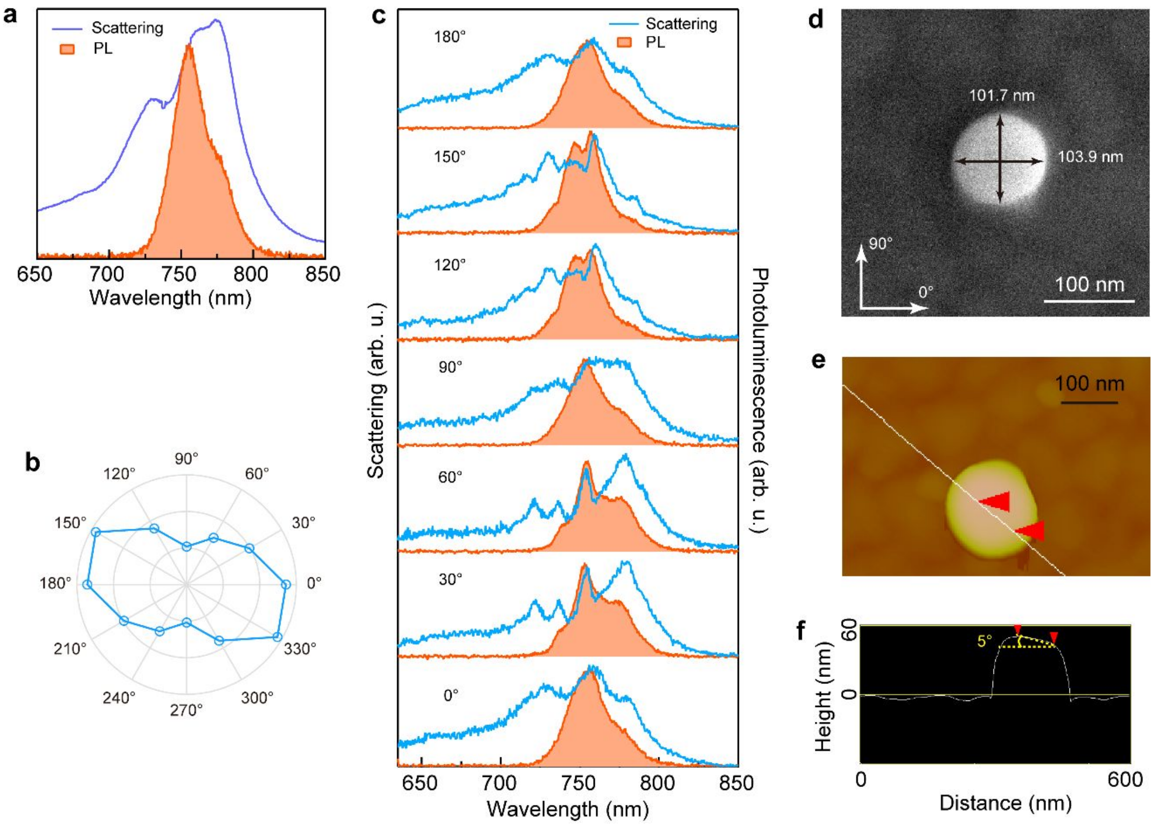

Figure S9. Optical and structural characterization of a typical WSe_2-monolayer sandwiched NDoM structure. (a) Scattering and PL spectra of a NDoM cavity without polarization resolution. (b) Scattering intensity as a function of the detection polarization angle under nonpolarized excitation. (c) Polarization-resolved scattering and PL spectra. The scattering spectra were acquired under the excitation of nonpolarized white light. The PL spectra were measured under the excitation of the linearly polarized laser at 633 nm. (d) SEM image of the NDoM cavity. (e) AFM image of the NDoM cavity. (f) Height profile extracted along the white line in (e), which reveals a ~5° tilting angle of the ND.

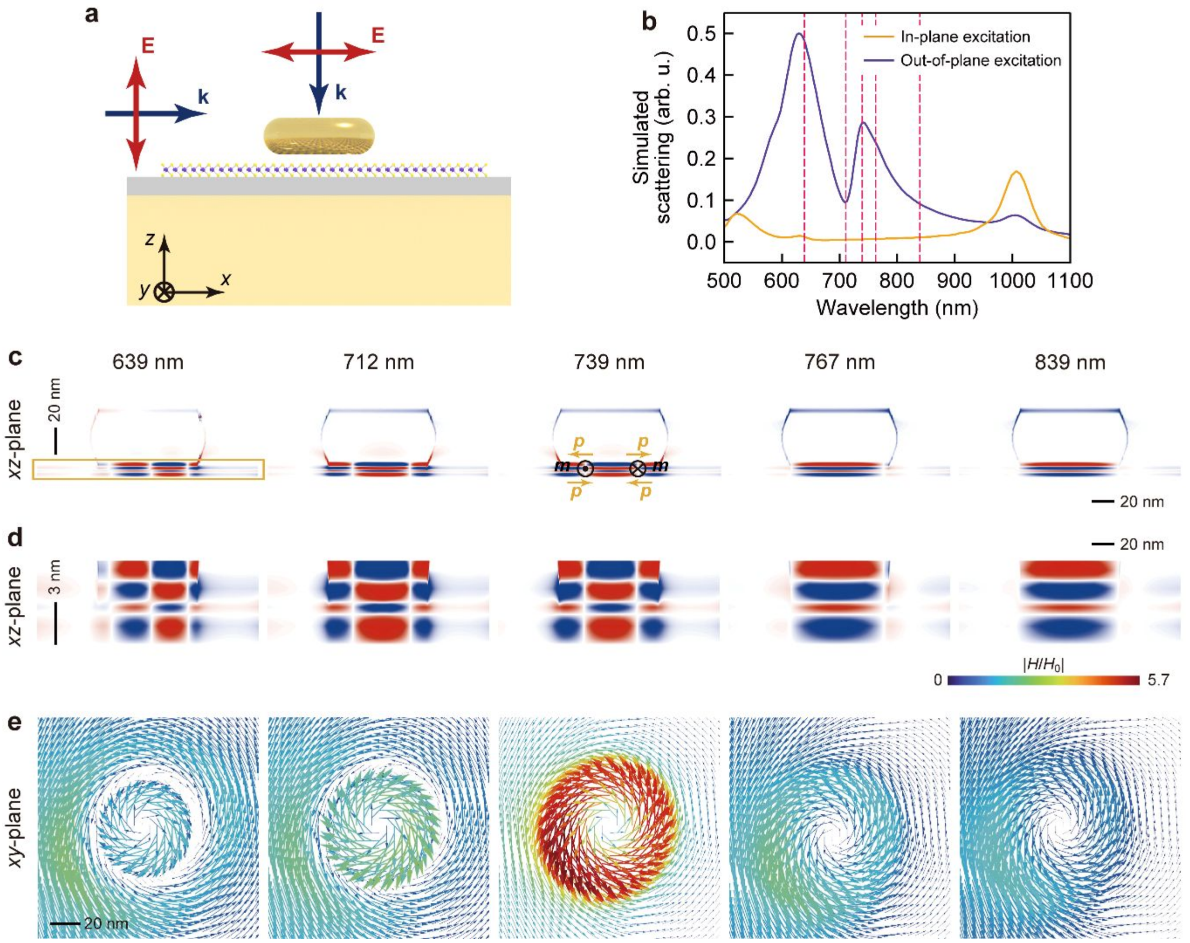

Figure S10. Charge distributions and magnetic vector field maps of the toroidal mode within −100 nm to +100 nm relative to the first-order toroidal mode peak wavelength. (a) Schematic of the NDoM cavity under in-plane or out-of-plane excitation. The nanodisk in the simulation model has a diameter of 103 nm and a thickness of 40 nm. We set the thickness of the Al_2O_3 layer to 1 nm in the simulations. (b) Simulated scattering spectra under in-plane and out-of-plane excitation. The dashed lines in the plot indicate the wavelengths of 639 nm, 712 nm, 739 nm, 767 nm, and 839 nm. (c) Charge distributions of the NDoM cavity at five wavelengths ranging from −100 nm to +100 nm relative to the peak wavelength under out-of-plane polarization excitation. The red and blue colors represent positive and negative charges, respectively. The simulation wavelength for each plot is indicated at the top. The orange box in the leftmost plot

highlights the gap between the Au nanodisk and the Au film. (d) Zoomed-in charge distributions of the gap in (c) at the five wavelengths. (e) Distribution contours of the induced magnetic field in the WSe_2 monolayer of the NDoM structure.

Supplementary Note 1. Two-coupled-harmonic-oscillator model

The polariton dispersion was modeled using a two-coupled-harmonic-oscillator model

$$\begin{pmatrix} E_{\text{pla}} + i\frac{\hbar\gamma_{\text{pla}}}{2} & g \\ g & E_{\text{bri}} + i\frac{\hbar\gamma_{\text{bri}}}{2} \end{pmatrix}\begin{pmatrix} \alpha \\ \beta \end{pmatrix} = E_{\pm}\begin{pmatrix} \alpha \\ \beta \end{pmatrix} \tag{1}$$

where E_{pla} is the uncoupled plasmon energy, E_{bri} is the uncoupled bright A exciton transition energy, γ_{pla} is the plasmonic dissipation rate, γ_{bri} is the excitonic dissipation rate, and g is the coupling strength. α and β are the Hopfield coefficients, which satisfy the condition $|\alpha|^2 + |\beta|^2 = 1$. The uncoupled plasmon energy E_{pla} was determined according to $E_{\text{pla}} = E_+ + E_- - E_{\text{bri}}$, where E_+ and E_- are the energies of the upper and lower branches extracted from the measured scattering spectra. E_{bri} was taken to be 1.655 eV according to the PL measurements. The polariton dispersion was fitted with the simplified expressions for the eigenvalues

$$E_{\pm} = \frac{E_{\text{pla}} + E_{\text{bri}}}{2} \pm \sqrt{g^2 + \frac{1}{4}\delta^2} \tag{2}$$

$$g = \sqrt{(E_{\text{bri}} - E_-)(E_+ - E_{\text{bri}})} \tag{3}$$

where $\delta = E_{\text{pla}} - E_{\text{bri}}$ is the detuning energy between the plasmons and excitons. $\hbar\Omega_{\text{R}} = 2g$ is the Rabi splitting at $E_{\text{pla}} = E_{\text{bri}}$. For the PL dispersion, the Rabi splitting was fitted to be 21.28 ± 1.94 meV.

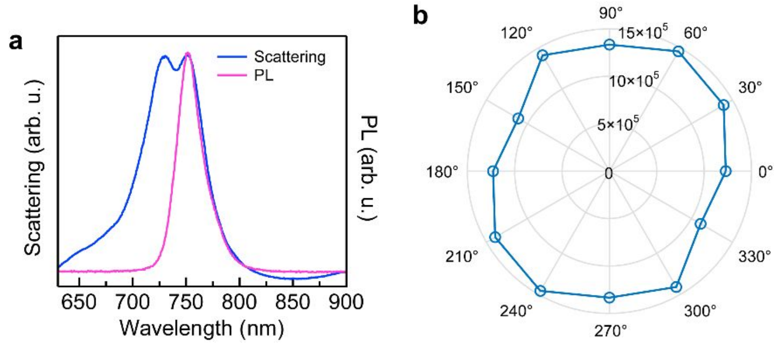

Figure S11. Scattering and PL measurements of a NDoM cavity with the toroidal plasmon resonance aligned with the WSe_2 bright A exciton emission energy. (a) Scattering and PL spectra of the NDoM cavity without polarization resolution. (b) Scattering intensity as a function of the detection polarization angle of the NDoM cavity in (a) under unpolarized excitation. The linear polarization degree was determined to be 10.1 ± 9.3% through fitting by $I = A\sin^2(\theta + \phi) + C_0$.

Table S1. Valley Coherence Performance Reported in Other Works

Material	Structure	Temperature	Degree of PL linear polarization	Reference
WSe_2 monolayer	Without any cavity	4 K	15%	*Phys. Rev. Lett.* **2016**, *117*, 187401
WSe_2 monolayer	Without any cavity	4.2 K	15%	*Nat. Commun.* **2018**, *9*, 4797
WSe_2 monolayer	DBR cavity	4.2 K	40%	*Nat. Commun.* **2018**, *9*, 4797
MoS_2 monolayer	Metasurface	unspecified	9%	*Phys. Rev. Lett.* **2018**, *121*, 116102
WSe_2 monolayer	DBR cavity (*Q*-factor ~240)	298 K	9%	*Nat. Commun.* **2019**, *10*, 1513
WS_2 monolayer	Plasmonic sawtooth nanoslit array	298 K	Linear dichroism (LD, LD = $(I_{TE} - I_{TM})/(I_{TE} + I_{TM})$) 80% ($LD_{valley}$ = ~20%)	*Nat. Commun.* **2020**, *11*, 713
WSe_2 monolayer	DBR cavity	298 K	17%	*Nat. Commun.* **2021**, *12*, 6406
WSe_2 monolayer	Single Au-nanodisk-on-mirror cavity	298 K	12%	This work

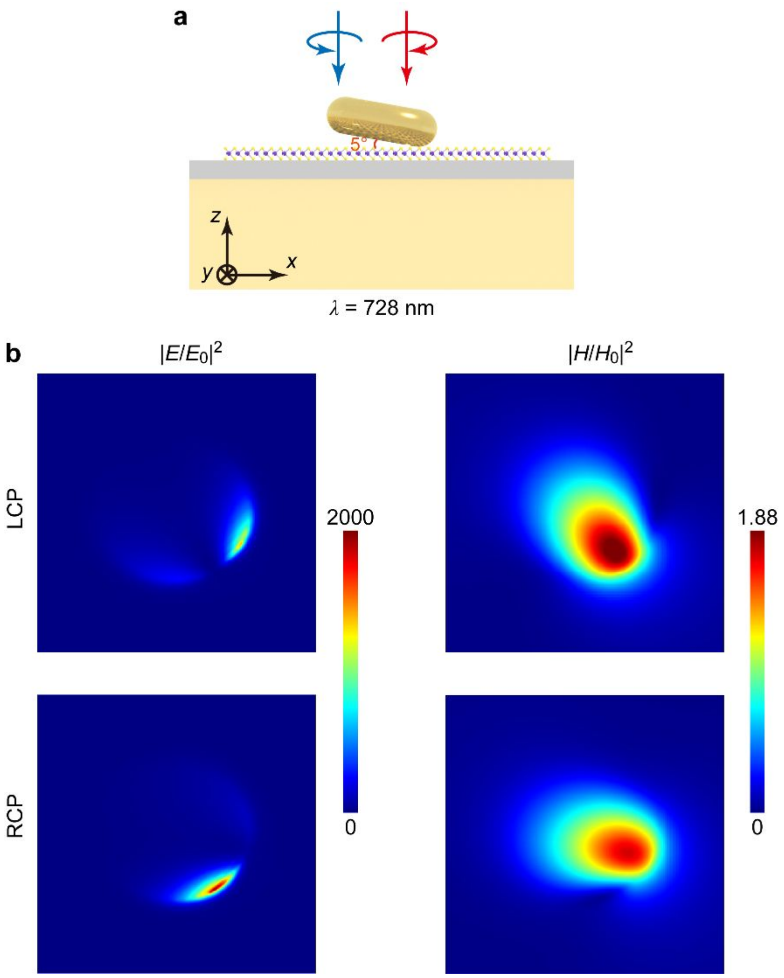

Figure S12. Chiral Purcell effect. (a) Schematic for a small tilt between the Au ND and the substrate that creates chirality. The ND in the model is rotated against axis *y* by 5°. The ND was modeled as an elliptic one with the long axis (along *x*) being 104 nm and short axis (along *y*) being 99 nm. (b) Electric field intensity enhancement $|E/E_0|^2$ (left column) and magnetic field intensity enhancement $|H/H_0|^2$ (right column) contours for the NDoM structure. The field enhancement contour was simulated at the center of the WSe_2 monolayer in the *xy* plane.

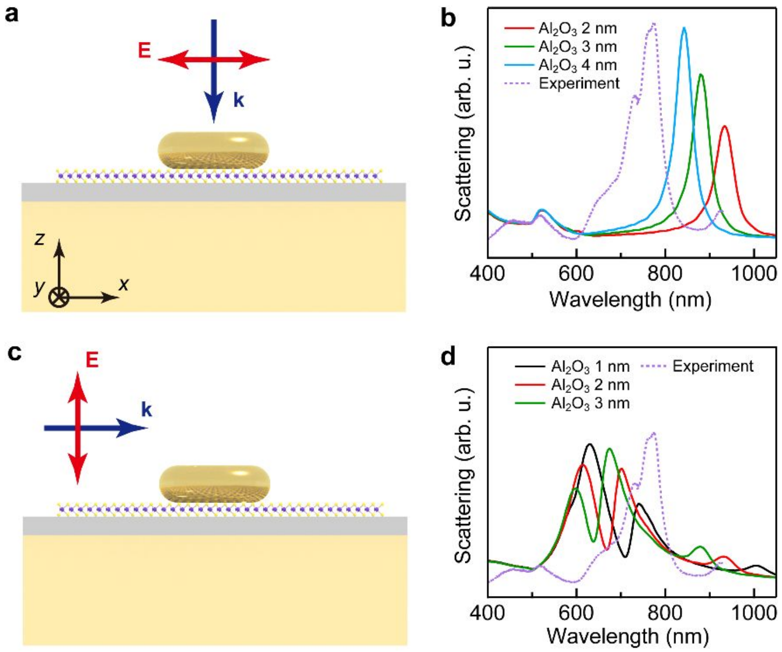

Figure S13. FDTD simulations of the scattering spectra of a WSe_2-monolayer-sandwiched NDoM cavity under in-plane and out-of-plane excitation. (a) Schematic of the NDoM cavity under in-plane excitation. (b) Measured and simulated scattering spectra using the model in (a) with varying Al_2O_3 spacer thicknesses. (c) Schematic of the NDoM cavity under out-of-plane excitation. (d) Measured and simulated scattering spectra using the model in (c) with varying Al_2O_3 spacer thicknesses.

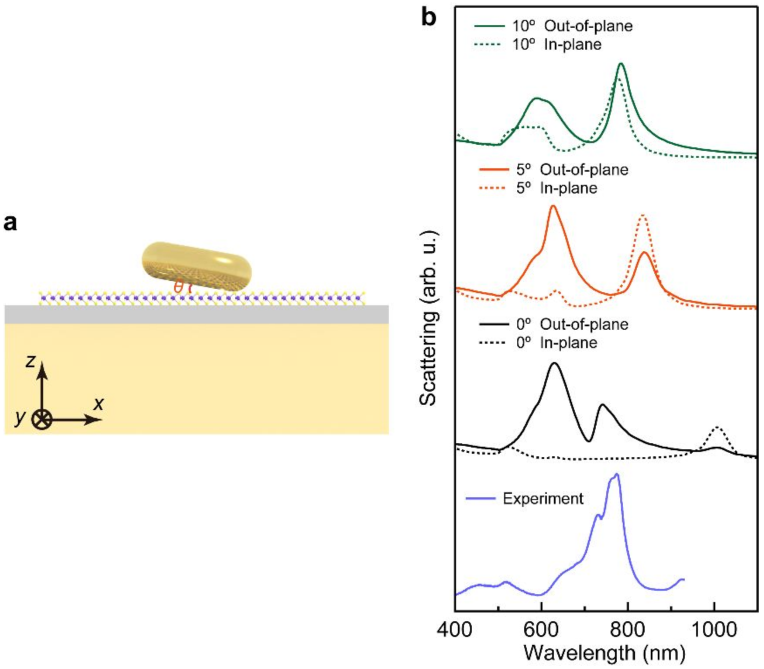

Figure S14. Effect of ND tilting on the scattering spectrum. (a) Schematic of a NDoM cavity constructed out of a tilted Au nanodisk. (b) Measured and simulated scattering spectra under both in-plane and out-of-plane excitation with different tilting angles θ. We set the thickness of the Al_2O_3 layer to 2 nm in the simulations.

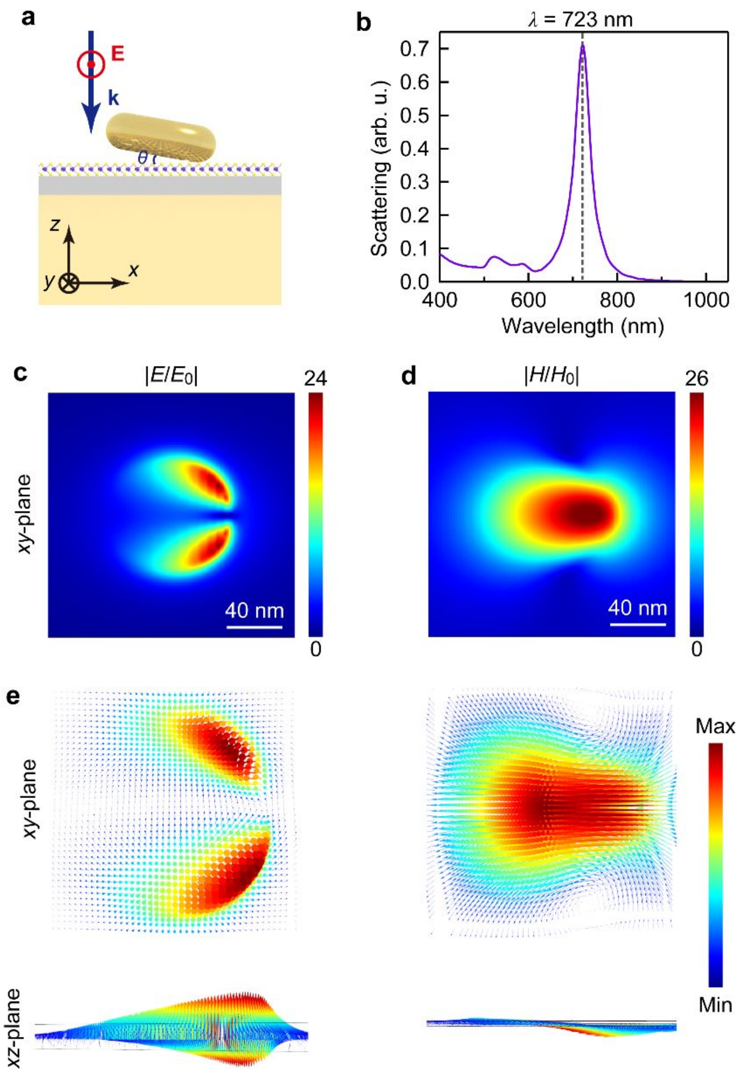

Figure S15. In-plane magnetic mode. (a) Schematic illustrating the simulation model for the NDoM structure excited by an in-plane electric field with its polarization direction parallel to the tilting axis. (b) Simulated scattering spectrum. (c,d) Simulated electric field enhancement and magnetic field enhancement contours at 723 nm, respectively. (e) Electric (left column) and magnetic (right column) field vectors at 723 nm. All the field distributions were obtained at the *xy*-plane located at the center of the WSe_2 monolayer.

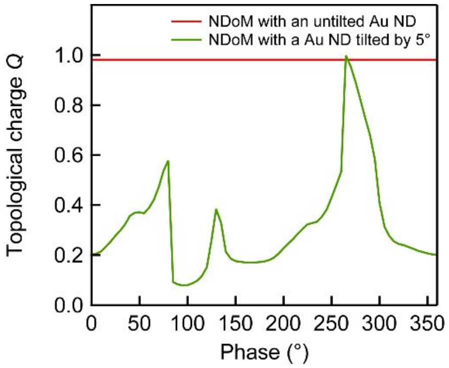

Figure S16. Calculated topological charges as a function of the electric field phase. The considered NDoM cavities had an untitled Au ND and a Au ND tilted by 5°. We carried out electromagnetic simulations on the WSe_2-monolayer-sandwiched NDoM cavity with a disk diameter of 103 nm and a thickness of 40 nm. The WSe_2 monolayer and the Al_2O_3 layer thicknesses were set at 1 nm and 2 nm in the simulation model, respectively. For the tilted-ND case, the ND was tilted against the *y* axis by 5°. The incident wave for the untilted case is out-of-plane polarized. The incident wave for the tilted case is in-plane polarized, including both *x*- and *y*- polarization directions. We calculated the topological charges for the simulated electric fields at 739 nm and 747 nm for the tilted and untilted cases, respectively, with varied phases.
